# Perceived sound localization abilities in blind individuals

**DOI:** 10.1371/journal.pone.0342118

**Published:** 2026-02-06

**Authors:** Prachi Agrawal, Haley Cihunka, Joseph Paul Nemargut, Walter Wittich, Chris Bradley, Yingzi Xiong

**Affiliations:** 1 Lions Vision Research and Rehabilitation Center, Wilmer Eye Institute, Johns Hopkins University, Baltimore, Maryland, United States of America; 2 Master of Science in Physician Assistant Studies Program, University of Dubuque, Dubuque, Iowa, United States of America,; 3 École d’optométrie, Université de Montréal, Montréal, Quebec, Canada; Texas Christian University, UNITED STATES OF AMERICA

## Abstract

**Purpose:**

There is extensive research on sound localization performance of blind individuals in laboratory settings. However, less is known about their spatial hearing experiences in more complex everyday environments. This study aims to assess the perceived sound localization abilities in blind individuals in common everyday tasks, which is a critical determinant of confidence and engagement in such tasks (e.g., independent travel).

**Method:**

Fifty-eight adults with total or near-total blindness, including 28 with self-reported normal hearing and 30 with self-reported hard of hearing participated in this study. They completed the Dual Sensory Spatial Localization Questionnaire (DS-SLQ), a recently developed instrument that evaluates perceived difficulty in everyday localization tasks using vision and hearing. Properties of the DS-SLQ for blind individuals, including whether its items measure a single attribute (i.e., unidimensionality) and whether item difficulties match blind individuals’ perceived abilities (i.e., targeting), were validated using Rasch analysis. Multivariable regression models and correlation analysis evaluated the impacts of the onset of blindness, residual vision, hearing status, onset of hearing loss, use of hearing aids, and echolocation skills on perceived sound localization abilities.

**Results:**

The DS-SLQ showed excellent targeting and good unidimentionality for blind individuals. Individuals with later onset of blindness reported significantly lower perceived sound localization abilities. The presence of hearing loss, particularly early onset, was associated with further reductions in perceived sound localization ability. Those who reported higher echolocation skills also reported better perceived sound localization abilities, regardless of their hearing status. Residual vision and use of hearing aids were not associated with perceived sound localization abilities.

**Conclusion:**

Beyond frequently studied vision factors, hearing loss and its onset further affect perceived sound localization abilities and the likelihood of an individual being an echolocator. It is critical to consider hearing status in the rehabilitation of blind individuals for maintaining and enhancing spatial localization skills.

## Introduction

The ability to perceive and localize objects in space is fundamental for navigating the environment and performing daily activities. For people with normal vision, vision plays a dominant role in spatial tasks. However, blind individuals who have no form vision rely heavily on their hearing to compensate for their vision loss. Developing spatial hearing skills has been an important part of the rehabilitation strategies for these individuals in locating objects beyond the reach of an arm or white cane [1,2].

Extensive research has focused on the spatial hearing abilities of blind individuals. A general finding is task-specific superiority of blind individuals compared to normally sighted individuals when locating simple sounds in laboratory conditions [e.g., 3–5]. However, few studies have investigated the performance of blind individuals in real-world sound localization tasks with a wide variety of sound features and environments. Existing studies focus primarily on street crossing and consistently find that blind individuals perform worse than those with normal vision and make more risky decisions [6–8]. These results indicate the importance of understanding spatial hearing abilities of blind individuals in complex real-world sound localization tasks.

An individual’s self-perception of their spatial hearing ability is also critical, as it influences their willingness to engage in independent travel or perform tasks such as street crossing. A blind individual with strong sound localization skills may report significant difficulty in such tasks, which could lead them to avoid or limit certain activities. On the other hand, a blind individual with poor sound localization skills who is overconfident in their skills may make risky decisions. Both perceived and actual ability are essential components in the rehabilitation for blind individuals, such as Orientation & Mobility training [9–11]. However, in routine clinical settings, obtaining objective measures of spatial localization in real-world contexts is often resource-intensive and not always feasible. Self-reported ability is easier to obtain and provides initial information on an individual’s functional status. Currently, there is no systematic investigation into the perceived sound localization abilities of blind individuals in real-world contexts.

The primary goal of this study is to evaluate perceived sound localization abilities and factors that contribute to perceived sound localization ability in blind individuals. Several factors are of interest based on previous laboratory studies. Earlier onset of blindness has been reported to be associated with better sound localization abilities than later onset [12]. The impact of residual vision is less clear, with some studies reporting residual vision in early life to be crucial for developing sound localization abilities while others found the opposite [13]. Another factor is blind individuals’ ability to use echolocation. While echolocation can take both active and passive forms, our investigations will focus on active echolocation, i.e., using echoes from self-generated sounds such as mouth clicks or cane taps to sense the environment. Echolocation is thought to complement external sound localization, as both rely on interpreting auditory spatial cues. Individuals who develop active echolocation skills tend to exhibit better spatial awareness and object localization [14,15].

Blindness is also often accompanied by hearing loss, due to congenital conditions affecting both senses and it can occur because of aging. Combined vision and hearing loss, also known as Dual Sensory Loss (DSL), significantly reduces mobility and quality of life [16], and is linked to increase risk of depression, social isolation, and decreased functional independence [17,18]. DSL encompasses a wide range of pathologies and severities of vision and hearing loss [19]. In a recent study, we found that among individuals with varying levels of vision, those who were blind were most likely to restrict their independent travel due to hearing loss [20]. Despite these findings, the specific impact of hearing loss on perceived spatial hearing abilities in blind individuals remains unexplored. In addition to general hearing status, our study also investigates the impact of the onset of hearing loss, binaural hearing asymmetry -- since inter-aural time and level differences are critical cues for sound localization [21,22] -- as well as the use of hearing aids on perceived sound localization abilities.

To this end, we employed the Dual Sensory Spatial Localization Questionnaire (DS-SLQ), which has been validated to assess auditory and visual spatial localization abilities in individuals with low vision with and without hearing loss [23]. We examine the psychometric properties of the DS-SLQ in blind individuals and evaluate factors associated with their perceived sound localization abilities. This study is a critical component of our ongoing investigation comparing perceived and actual abilities of blind individuals in real-world sound localization tasks.

## Methods

### Participants

Fifty-eight participants were recruited from the Minnesota Laboratory for Low Vision Research, and the Envision Low Vision Rehabilitation Center. Inclusion criteria were age 18 years or older, self-reported total or near-total blindness with no form vision (i.e., no light perception, light perception, hand motion, or finger counting), and no cognitive impairments. This study was approved by the University of Minnesota Institutional Review Board (IRB number: STUDY00001360) and followed the Declaration of Helsinki (World Medical Association, [24]). Verbal consent was approved by the Institutional Review Board as part of this survey protocol. Consenting information was incorporated as the first section of the survey and read to the participants by the researcher. Participants can only proceed to the main survey after providing their verbal consent. The survey data was collected from 02/21/2022–07/14/2022.

Based on the level of self-reported residual vision, 34 participants were totally blind without light perception, and 24 participants were near-total blind with light perception, finger counting or hand motion. Out of all the 58 participants, 28 self-reported normal hearing (Blind-NH), and 30 participants self-reported with hearing loss. Because of the broad range of severity and onsets of hearing loss in our participants, we will use the term “hard of hearing” (Blind-HH) when referring to this participant group, per National Association of the Deaf recommendations [25].

### Dual Sensory-Spatial Localization Questionnaire (DS-SLQ)

The DS-SLQ includes descriptions of 35 everyday tasks that require localization of common objects around them like people or cars. For each task, participants responded to how difficult it was to complete each task with their vision alone or hearing alone based on their everyday experiences, using ratings from 1–5 with 1 being very easy and 5 being very difficult. They could respond “Not applicable” if unfamiliar with the tasks or situation posed in the question. The DS-SLQ was implemented as an online survey tool on Qualtrics and delivered through zoom videoconferencing or a phone call according to participant’s preference. More details of the development and validation of DS-SLQ can be found in Xiong et al. [23].

When administering the DS-SLQ to blind participants in the current study, vision ratings for each task were obtained for those with residual vision (e.g., light perception, hand motion, or finger counting), while were omitted for individuals without light perception. Responses were registered on Qualtrics by the researcher.

Prior to completing the DS-SLQ, participants first completed screening questions asking about their demographical information, including sex at birth and age, onsets for vision and/or hearing loss, rehabilitation history such as Orientation & Mobility training, and binaural asymmetry and use of hearing aids for those with hearing loss. Further, participants were asked to provide their history of using echolocation. Echolocation was defined for participants as: *“The ability to sense your environment and objects surrounding you by listening to the echoes returned when making a sound such as mouth clicks, finger taps, cane taps, etc.”.* Participants who self-reported as echolocators were further asked to rate their expertise in echolocation from 1–5, with the rating 1 being when they were able to echolocate but not very well, and rating 5 being an expert at echolocating. The entire survey took about 50 minutes to 1.5 hours in total.

### Data analysis

All data analysis was performed using R statistical software (R Core Team [26]). Responses from the DS-SLQ were analyzed using the method of successive dichotomizations (MSD), using the R package ‘msd’ (Bradley & Massof [27]). MSD yields two primary outcomes: the *item measure*, indicating the difficulty of each task (higher values reflect more difficult tasks), and the *person measure*, representing an individual’s perceived spatial ability across all tasks (higher values indicate better perceived ability). A key feature of the DS-SLQ is that each task includes paired vision and hearing ratings, allowing assessment of spatial localization using either sense. To directly compare the difficulty of completing each task via vision versus hearing, a combined MSD analysis was conducted to align vision and hearing item difficulties on the same scale. The calibrated item measures were then anchored (fixed) in two separate MSD analyses, one for vision-only ratings and one for hearing-only ratings, to estimate perceived spatial abilities for each modality independently (see Xiong et al. [23]).

To evaluate the psychometric properties of the DS-SLQ for blind individuals, we examined two key psychometric properties: unidimensionality and targeting. Unidimensionality refers to a single underlying attribute (e.g., perceived auditory spatial ability in this case) that is being measured by the items of an instrument. We examined the information-weighted mean square fit (infit) statistics — acceptable infit values were within a range of 0.5–1.5, in accordance with COSMIN recommendations [28] — and conducted a principal component analysis to estimate the variance explained by the first principal component. Targeting refers to the degree to which the distribution of items measures matches that of the person measures. Poorly targeted instruments may show ceiling or floor effects, leading to highly imprecise estimates of person measures. Targeting was assessed for the DS-SLQ by comparing the distributions of item measures and person measures, to examine whether the perceived difficulty of the spatial localization tasks match the perceived spatial localization abilities in our sample of blind individuals.

Descriptive statistics summarized key characteristics of participants, including age, onset of vision and hearing loss, use of hearing aids, hearing asymmetry, echolocation skills, and Orientation & Mobility training history. Multivariable regression models were constructed to explore factors associated with perceived sound localization abilities (as reflected by DS-SLQ hearing person measures). An ANOVA was used to obtain the significance of each factor. Effect size was estimated using *η*^2^ or partial *η*^2^.

## Results

### Participant characteristics

Table 1 summarizes the characteristics of participants in the Blind_NH and Blind_HH groups. The Blind_HH group was slightly older than the Blind_NH group (51.6 vs 59.5 yrs, F(1,55) = 4.46, p = 0.039, *η*^2^ = 0.08). Most of the participants had early onset vision loss, with an average onset of 8 yrs for the Blind_NH group and 10.5 yrs for the Blind_HH group. The primary causes for the blindness include retinitis pigmentosa and retinopathy or prematurity. Most of the participants in the Blind_HH group had later onset of hearing loss than their vision loss (mean difference = 26.4 yrs, F(1, 56) = 22.17, p < 0.001, *η*^2^ = 0.28). Only four of the Blind_HH participants reported hearing loss from birth due to the same medical condition as their vision loss (3 with Usher Syndrome, 1 with Marshall Syndrome).

**Table 1:** Characteristics of participants in the Blind_NH and Blind_HH groups.

	Blind_NH	Blind_HH
N	28	30
Age, mean (SD), years	51.6 (11.5)	59.5 (15.9)
Gender, female, N (%)	19 (67.9%)	14 (46.7%)
Vision, no light perception, N (%)	14 (50.0%)	20 (66.7%)
Onset of vision loss, mean (SD), years	8.0 (12.3)	10.5 (16.3)
Onset of hearing loss, mean (SD), years	NA	37.4 (26.4)
Hearing loss asymmetry, yes, N (%)	NA	21 (70%)
Hearing aids, yes, N (%)	NA	21 (70%)
Echolocation, yes, N (%)	22 (78.6%)	16 (53.3%)
Orientation & Mobility training, yes, N (%)	27 (96.4%)	28 (93.3%)

In total, 96.4% and 93.3% of the participants in the Blind_NH and Blind_HH groups have received O&M training, respectively. In the Blind_HH group, 70% of the participants reported one ear to be better than the other, and 70% reported as hearing aids users.

### Psychometric validity of DS-SLQ for blind individuals

**Fig 1:**
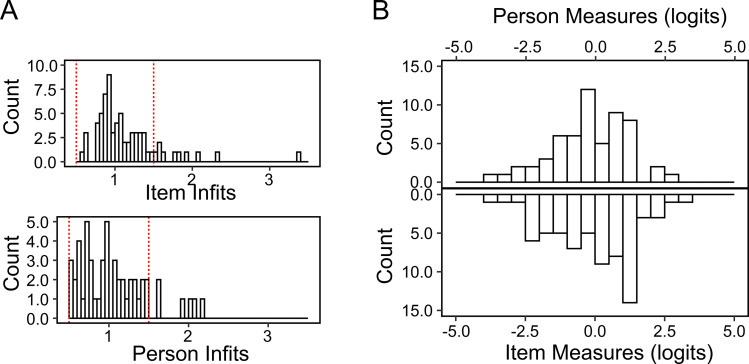
Infits and targeting of the DS-SLQ. **(A)** Histogram showing the distribution of item and person infit statistics. Most items and persons have infit values between 0.5 and 1.5, indicated by the red dash lines. **(B)** Comparison of the range of person measures (upper panel) and item measures (lower panel) on the same logit scale.

Figs 1A illustrates the distributions of item and person infits for blind participants in our sample. Approximately 84% of the item measures and 78% of the person measures fall within the desired infit range of 0.5–1.5, indicating acceptable unidimensionality of the DS-SLQ in assessing perceived sound localization abilities among blind individuals. Principal component analysis on the raw ratings further confirmed the unidimensionality, with the first principal component explaining 66.5% of the variances.

Fig 1B illustrates “targeting” of DS-SLQ for blind participants by showing the distributions of the person measures (upper panel) and item measures (lower panel). Item measures ranged from −3.6 to 3.1 logits, while the person measure ranged from −4.0 to 2.9 logits. When comparing these distributions, 98% of the person measures landed within the range of item measures, demonstrating excellent targeting of DS-SLQ for blind individuals.

### Factors associated with perceived sound localization abilities in blind individuals

To understand factors associated with perceived sound localization abilities, in a multivariable regression model across all participants we treated hearing person measures as the dependent variable, age (in years), onset of blindness (in years), residual vision status (residual vision vs. no light perception), and hearing status (NH vs. HH) as independent variables. Although we were also interested in O&M training, the very high prevalence of O&M training history in both groups prevented us from including this factor.

**Fig 2:**
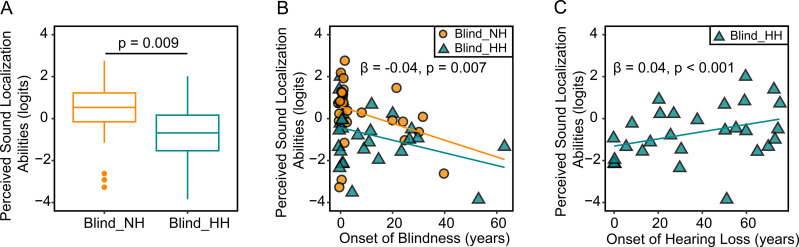
Perceived sound localization abilities across hearing and vision status. **(A)** Comparison of hearing person measures from the DS-SLQ between blind individuals with normal hearing (Blind_NH) and those with hearing loss (Blind_HH). **(B)** Scatter plot illustrating the relationship between perceived sound localization abilities and onset of blindness in blind individuals. **(C)** Relationship between perceived sound localization ability and onset of hearing loss in blind individuals. In each plot, orange and cyan colors represent Blind_NH and Blind_HH groups, respectively. Regression lines are provided in the scatter plots.

The result showed that later onset of blindness (β = −0.04, F (1, 52) = 8.03, p = 0.007, partial *η*^2^ = 0.13) and presence of self-reported hearing loss (F (1, 52) = 7.48, p = 0.009, partial *η*^2^ = 0.13) were associated with significantly lower perceived sound localization abilities. Because the onsets of blindness clustered around 0 years old, we repeated this analysis on those who acquired blindness after birth and found the same effect of the onsets (β = −0.04, F (1, 26) = 6.18, p = 0.020, partial *η*^2^ = 0.19). There was no interaction between the onset of blindness and hearing status, suggesting that the effects of onsets of blindness were uniform across the blind individuals. Figs 2A and 2B visualized the distribution of sound localization abilities across these two factors, respectively.

Next, we focused on individuals with hearing loss, asking whether the onsets of hearing loss and use of hearing aids played additional role in perceived hearing spatial abilities. In a multivariate regression model on the Blind_HH group, we again treated hearing person measures as the dependent variable, age, onset of blindness, residual vision status, as well as onset of hearing loss (in years), perceived binaural asymmetry (yes or no), and use of hearing aids (yes or no) as independent variables. The result again demonstrated a significant effect of the onset of blindness (β = −0.04, F (1, 20) = 6.99, p = 0.016, partial *η*^2^ = 0.26); in addition, earlier onset of hearing loss was associated with poorer perceived sound localization abilities (β = 0.04, F (1, 20) = 12.16, p = 0.002, partial *η*^2^ = 0.38) (Fig 2C). Perceived binaural asymmetry or use of hearing aids was not a significant factor.

### Echolocation and its relationship with perceived sound localization abilities

**Fig 3:**
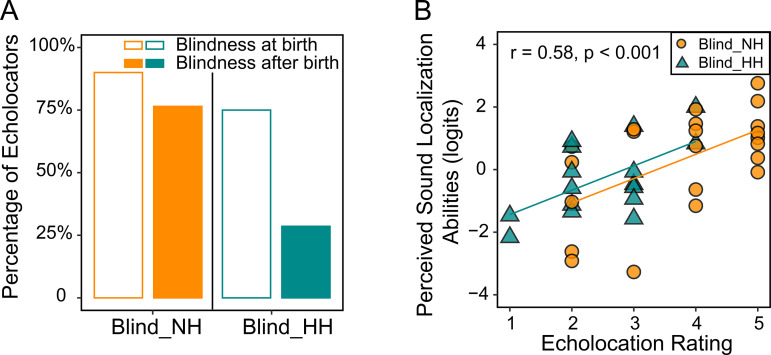
Echolocation and perceived sound localization abilities in blind individual. **(A)** Proportion of echolocators among Blind_NH and Blind_HH participants. **(B)** Scatter plot showing the correlation between perceived sound localization abilities and echolocation rating. Regression lines are also provided in the scatter plot. In both plots, the orange and cyan colors represent Blind_NH and Blind_HH groups, respectively.

We considered echolocation as a separate factor as it is a form of localization using self-generated sounds, different from locating external sounds that DS-SLQ intends to measure. In total, 66% of the blind participants self-reported to be echolocators. Participants with later onset of blindness (β = −0.07, χ² (1) = 9.27, p = 0.002) and presence of self-reported hearing loss (χ² (1) = 6.71, p = 0.010) were found to be less likely to be echolocators. It is interesting to note the similar effects of these two factors on DS-SLQ measured sound localization as mentioned above. In Fig 3A, we summarized the percentages of echolocators in the Blind_NH and Blind_HH groups, stratified by whether the onset of blindness was at birth.

Among participants who self-reported as echolocators, we further explored the associations between self-rated echolocation expertise and the perceived sound localization abilities measured by DS-SLQ. As shown in Fig 3B, there was a significant correlation between these two measures (r = 0.58, p < 0.001), independent of hearing status.

### Perceived visual localization abilities in blind individuals with residual vision

Amongst the 24 participants with residual vision who self-reported light perception, hand motion, or finger counting, 16 gave valid vision ratings (9 from Blind_NH and 7 from Blind_HH groups, respectively) on at least 2 of DS-SLQ tasks. Vision person measures were thus obtained from these 16 participants, allowing a more detailed description of the perceived utility of their residual vision in visual localization. We conducted the following exploratory analysis, due to the small sample of available vision measures.

**Fig 4:**
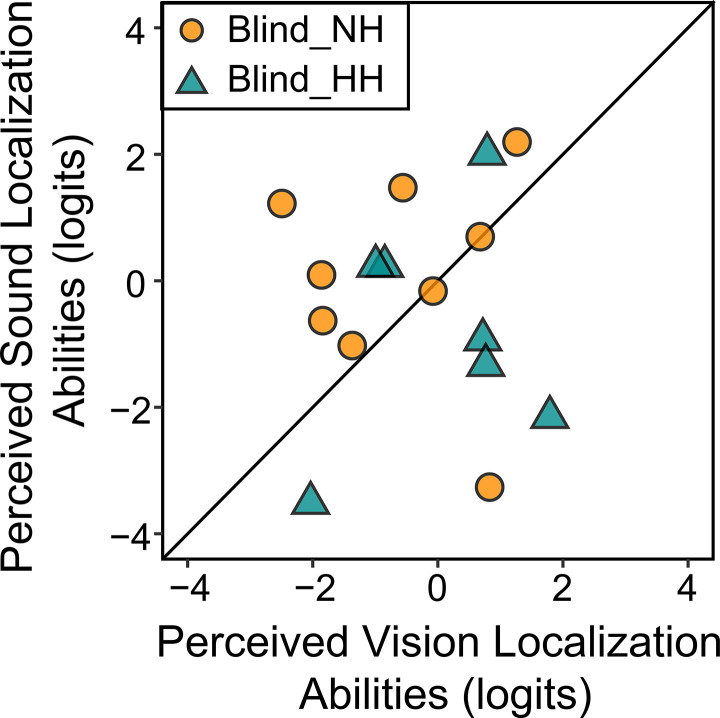
Comparison of perceived sound and visual localization abilities in blind participants. The scatterplot shows a relationship between perceived sound localization abilities and perceived vision localization abilities (both in logits). The diagonal line represents the equality line, where equal perceived localization abilities in both modalities would lie. Orange circles represent Blind_NH and cyan triangles represent Blind_HH.

Fig 4 illustrates the comparison of perceived vision and hearing localization abilities for these 16 individuals, with dark orange representing the Blind_NH group and dark cyan presenting the Blind_HH group. There was no significant correlation between the vision and hearing abilities in either group. When comparing the two localization abilities, more participants in the Blind_NH had higher hearing ability than vision, while more participants in the Blind_HH had higher vision abilities than hearing, suggesting that even very limited vision by clinical standard can play a critical role in blind individuals, especially in the presence of co-occurring hearing loss.

## Discussion

This study aimed to investigate factors affecting perceived sound localization abilities in blind individuals with and without concurrent hearing loss. We found that individuals with earlier onset of blindness reported higher perceived sound localization abilities. Presence of hearing loss reduced perceived sound localization abilities, especially for those with an early onset of hearing loss.

Although localization of external sound and echolocation of self-generated sound are very different tasks, we found interesting associations between the two. A blind individual with early onset of blindness is also more likely to be an echolocator and report superior echolocation abilities than those with later onset of blindness. This effect is unlikely to be explained by unequal access to Orientation & Mobility training, as almost all participants we tested have received systematic Orientation & Mobility training. This effect can’t be explained by a reduced ability to learn echolocation in adulthood either, as previous study has reported echolocation as a learnable skill by adults through training [14,29]. A more plausible explanation might be a reduced endorsement of echolocation skill when blindness is acquired later in life.

Echolocators with higher self-reported echolocation skills also reported higher sound localization abilities in DS-SLQ, even in the presence of hearing loss. It is possible that some individuals were overall more positive while subjectively rating their own abilities, but previous laboratory spatial localization studies have also shown a positive association between echolocation skills and performances in simple sound localization tasks (e.g., space bisection task) [15]. Our finding hints at the possible value of echolocation as a compensatory strategy to mediate the impact of hearing loss. However, objective measurements and comparisons of sound localization and echolocation performance are needed to quantify the true relation between these two tasks in real-world scenarios and the impacts of hearing loss on both.

Contemporary hearing rehabilitation primarily focuses on hearing aids prescriptions, which has gained great success in facilitating speech perception. In our recent study surveying impact of hearing loss among blind individuals, we found significantly lower satisfaction rate with using hearing aids for spatial hearing (see Reed et al. [20]). In our current study, we again did not find a positive impact with use of hearing aids for sound localization. As technology is advancing with increasing interest in the spatial hearing domain, it is crucial for Orientation & Mobility specialists to work with audiologists in the selection and fitting of the hearing aids that meet their clients’ needs for preserving spatial hearing.

We used our previously developed Dual Sensory Spatial Localization Questionnaire (DS-SLQ) in the current investigation, instead of other instruments such as the Speech, Spatial and Qualities of Hearing scale (SSQ) that specifically focuses on hearing [30]. DS-SLQ was developed with a unique focus on concurrent vision and hearing loss, allowing direct comparison of the residual vision and hearing abilities. Notably, using DS-SLQ, we found that even individuals with very low residual vision ranging from finger counting to light perception still reported measurable use of their residual vision in spatial tasks, outperforming their sound localization in some individuals with DSL. This retained visual input may be especially valuable for those with concurrent hearing loss, where any remaining sensory information can support orientation and mobility.

Perceived abilities, though subjective, offer valuable insight into how individuals interpret their own functionality and confidence navigating real-world environments. For instance, two individuals with similar objective sound localization performance may differ significantly in travel independence depending on their self-assessed competence. Several studies reported discrepancies between subjective report and objective performance. For example, Turano et al., reported a dissociation between perceived and actual independent mobility in patients with retinitis pigmentosa [31], and West et al., reported lack of correlation between perceived and functional performances in older adults with vision loss [32]. Subjective assessments often capture psychological factors such as confidence, fear, and motivation, which objective tests may miss [9,10]. Thus, understanding perceived spatial hearing is not just complementary to objective testing, it is essential for comprehensive rehabilitation planning.

Due to our focus on perceived abilities and the items on DS-SLQ being complex real-world tasks, it is difficult to draw quantitative comparisons between our findings and previous behavior studies. In Table 2, we summarize representative behavioral research that measured sound localization or echolocation performance in blind individuals. Most studies had relatively small sample size for comprehensive analysis on onset, but one study reported early blind individuals showing more superior horizontal sound localization than later-blind individuals [12], which is consistent with the impact of onset of blindness found in our study. Across studies, the superiority of sound localization appears to be task dependent. The real-world tasks on DS-SLQ encompass many of these aspects of spatial hearing, including direction, distance, horizontal, vertical localization etc. It is possible that these spatial hearing tasks all contribute to an individual’s perceived abilities measured by DS-SLQ. Lastly, all existing studies only included participants with normal hearing. Our study, although subjective, is among the first to assess perceived sound localization abilities in blind individuals that are hard-of-hearing.

**Table 2:** Summary of representative behavioral studies on sound localization in blind individuals.

Citation	Sample^a^	Onset	Hearing	Task	Conclusion
Lessard et al., 1998 [33]	8 totally blind subjects; 29 controls	Congenital or early blindness	Assumed normal hearing	Horizontal direction and distance judgement under monaural and binaural listening.	Early blind individuals localized sounds better than sighted participants, especially under monaural conditions.
Zwiers et al., 2001 [34]	6 blind individuals; 7 controls	Congenital or early blindness	Normal hearing	Vertical sound localization	Blind participants showed reduced elevation localization accuracy
Voss et al., 2004 [12]	23 blind individuals; 10 controls	Early (n = 14) and late (n = 9) blindness	Normal hearing	Horizontal direction judgement	Early blind individuals exhibited superior localization accuracy, while late-blind performed similarly to controls.
Ashmead et al., 2005 [6]	6 blind individuals; 6 controls	Not reported.	Normal hearing	Real-world street crossing decision-making.	Blind pedestrians made less safe street-crossing decisions than sighted pedestrians.
Gougoux et al.,2005 [35]	12 blind individuals; 7 controls	Early blind before puberty	Normal hearing	Horizontal direction judgement under monaural and binaural listening	Individuals with superior localization showed more activation of auditory cortex.
Teng et al., 2011 [36]	6 blind individuals; 4 controls	Mixture of early and late blindness.	Assumed normal hearing	Spatial acuity tasks using echolocation	Blind echolocators achieved remarkably high spatial resolution, outperforming sighted controls.
Hassan et al. 2012 [8]	10 blind individuals; 10 low vision; 12 controls	Not reported.	Normal hearing	Real-world street crossing decision-making using hearing only.	Blind pedestrians performed worse than the normally sighted or low vision subjects.
Guth et al., 2013 [7]	10 blind; 9 controls	5 congenitally blind, 5 late onset	Normal hearing	Real-world street crossing decision-making.	Blind participants’ judgments about when to cross were more frequently risky, especially when traffic volume was high.
Gori et al., 2014 [37]	9 blind individuals; 27 controls	Congenital blindness	Normal hearing	Spatial bisection, minimal audible angle, pointing, temporal bisection	Congenitally blind adults showed severe deficits in spatial bisection.
Vercillo et al., 2015 [15]	9 blind; 11 controls	Congenitally blind	Normal hearing	Space bisection and minimum audible angle task.	Blind echolocators showed superior auditory spatial localization compared to both blind non-echolocators and sighted controls.
Kolarik et al., 2017 [38]	9 blind individuals; 10 controls	Early blind	Normal hearing	Navigated obstacle using echolocation	Blind individuals were faster, more fluid, and accurate navigating around obstacles using sound.

^a^Only blind (no form vision) and control participants are summarized here.

Our study has several limitations. The robustness of our findings could benefit from a larger sample size. Due to the geographic diversity of our sample, hearing loss status was self-reported and categorized into binary groups. We obtained self-reported binaural asymmetry from our participants and did not find a significant association with perceived sound localization abilities. This finding is inconclusive as we did not have access to measured laterality of hearing loss (e.g., bilateral vs. unilateral or asymmetrical loss) or the number of hearing aids used (unilateral vs. bilateral fitting), which could influence sound localization performance. Future work should use audiometric testing and treat hearing loss as a continuous variable. Additionally, incorporating objective assessments of sound localization and echolocation in real-world tasks alongside subjective ratings would strengthen the understanding of their relationship and inform targeted rehabilitation strategies. Our ongoing work is comparing perceived abilities measured by DS-SLQ and actual performance in blind individuals with or without hearing loss.
